# Associated factors of mortality and morbidity in emergency and elective abdominal surgery: a two-year prospective cohort study at lacor hospital, Uganda

**DOI:** 10.1186/s12893-024-02433-z

**Published:** 2024-05-11

**Authors:** Ronald Okidi, Vanusa Da Consolacao Sambo, Isaac Okello, Doris Amarachi Ekwem, Solomon Ekwang, Fiddy Obalim, Willy Kyegombe

**Affiliations:** 1 Department of Surgery, Lacor Hospital, P.O. Box 180, Gulu, Uganda; 2 Faculty of Medicine, Gulu University, Gulu, Uganda; 3 Faculty of Medicine, Soroti University, Arapai, Uganda

**Keywords:** Abdominal surgery, Indication, Morbidity, Mortality

## Abstract

**Background:**

The mortality rate associated with open abdominal surgery is a significant concern for patients and healthcare providers. This is particularly worrisome in Africa due to scarce workforce resources and poor early warning systems for detecting physiological deterioration in patients who develop complications.

**Methods:**

This prospective cohort study aimed to follow patients who underwent emergency or elective abdominal surgery at Lacor Hospital in Uganda. The participants were patients who underwent abdominal surgery at the hospital between April 27th, 2019 and July 07th, 2021. Trained research staff collected data using standardized forms, which included demographic information (age, gender, telephone contact, and location), surgical indications, surgical procedures, preoperative health status, postoperative morbidity and mortality, and length of hospital stay.

**Results:**

The present study involved 124 patients, mostly male, with an average age of 35 years, who presented with abdominal pain and varying underlying comorbidities. Elective cases constituted 60.2% of the total. The common reasons for emergency and elective surgery were gastroduodenal perforation and cholelithiasis respectively. The complication rate was 17.7%, with surgical site infections being the most frequent. The mortality rate was 7.3%, and several factors such as preoperative hypotension, deranged renal function, postoperative use of vasopressors, and postoperative assisted ventilation were associated with it. Elective and emergency-operated patients showed no significant difference in survival (*P-value = 0.41*) or length of hospital stay (*P-value = 0.17*). However, there was a significant difference in morbidity (*p* < 0.001).

**Conclusion:**

Cholelithiasis and gastroduodenal perforation were key surgical indications, with factors like postoperative ventilation and adrenaline infusion linked to mortality. Emergency surgeries had higher complication rates, particularly surgical site infections, despite similar hospital stay and mortality rates compared to elective surgeries.

## Introduction

The mortality rate associated with open abdominal surgery is a significant concern for both patients and healthcare providers [1, 2]. Surgical care is extremely limited in Africa, with most of the population (93%) lacking access to it [1]. The procedure presents significant mortality challenges in Africa, possibly due to the diverse pathology of patients, scarce workforce resources and poor early warning systems for detecting physiological deterioration in patients who develop complications [3, 4].

Notably, patients older than 70 years old have a higher risk of adverse outcomes and postoperative complications [5–7]. Semulimi et al. (2022) revealed that patients undergoing emergency abdominal surgery face a 9.8 times higher risk of mortality compared to those undergoing elective procedures. Additionally, those needing ICU admission are 10.2 times more likely to experience complications and 18.2 times more likely to die compared to those not requiring ICU care. Notably, 21% of deaths following trauma abdominal surgery were attributed to haemorrhage [7, 8].

The most common indications for non-traumatic emergency abdominal surgery include peptic ulcer perforation, blunt abdominal trauma, and acute intestinal obstruction [9–11]. Modified Graham’s patch repair is the most commonly performed procedure, followed by ileostomy/colostomy placement [9]. Moreover, in trauma abdominal surgery, a study showed that the spleen was the most commonly injured organ in blunt trauma injuries, and the small intestine was more frequently injured in penetrating trauma. Blunt abdominal injuries were majorly caused by road traffic accidents and penetrating injuries by firearms, respectively [11, 12].

A study in Malawi showed a surprising gender imbalance among people needing surgery for peritonitis (a serious infection). Even without considering surgeries more common in men or women, like C-sections or hernias, nearly 70% of these patients were men [13].

Abdominal surgical emergencies are a significant part of surgeries performed in African hospitals, representing a frequency ranging from 20 to 22.7% [14]. Patients who undergo urgent abdominal surgery face up to five times higher risk of death when compared to those who undergo scheduled surgery [15]. According to a recent study, there is a high incidence of mortality following abdominal surgery, with infections being the main cause [14]. Abdominal surgery can result in various complications, including re-abdominal surgery, intestinal barrier malfunction, subsequent bacterial translocation, and postoperative sepsis, which can even lead to multiple organ failure [16]. Frequently observed postoperative complications encompass surgical site infections, chest infections, urinary tract infections, paralytic ileus, and delirium. Notably, surgical site infections consistently emerge as the most prevalent, with incidence rates ranging from 20 to 28.7% [9, 10, 17].

The surgical outcome depends on many factors, notably, patients who undergo primary abdominal closure during their initial hospitalization have shorter stays in intensive care and the hospital. However, longer hospital stays were found to result from failure of primary fascial closure, particularly in cases requiring more than four operations before primary closure was achieved [18, 19].

Lacor Hospital’s unique context of limited resources and diverse caseload demands context-specific knowledge. This 2-year study delves into factors influencing morbidity and mortality in both emergency and elective abdominal surgeries. Insights will directly guide the development of evidence-based interventions and policy changes, ultimately enhancing patient safety.

## Materials and methods

### Study setting

St Mary’s Hospital, Lacor, is a private, not-for-profit, church hospital situated in Gulu District, northern Uganda. The hospital has significant overseas support, and patient care is subsidized to fulfil its mission of serving the poorest patients to the highest standards possible. The hospital has a 482-bed capacity and is also a university teaching hospital for the Government University of Gulu, Faculty of Medicine.

### Study design

This research was carried out at the Department of Surgery, Lacor Hospital in Uganda, focusing on patients who underwent either emergency or elective abdominal surgery. The study involved a 30-day post-surgery follow-up period, encompassing both the time after hospital discharge and scheduled hospital visits. In cases where patients did not return to the hospital on their designated dates, we contacted them by phone to assess any morbidity or mortality experienced at home. Deaths occurring within 30 days of surgery were considered to be associated with the surgical procedure.

### The sampling technique

The sampling technique employed in this study utilized a consecutive simple sampling approach, wherein every patient presenting to the hospital with abdominal symptoms necessitating surgical intervention was recruited upon obtaining consent. These recruited patients were then prepared for surgery and subsequently followed up in the postoperative period.

### Participants, inclusion & exclusion criteria

We conducted a study at Lacor Hospital between April 27, 2019, and July 7, 2021. Our research team, trained by the hospital, enrolled participants who had undergone abdominal surgery. We recruited patients from both the accident and emergency unit and the surgery ward for emergency and elective cases, respectively. We included patients who met our comprehensive optimization and inclusion criteria and who agreed to participate after providing informed consent.

All emergency patients received 30 mL/kg body weight of intravenous crystalloids within the first three hours of presentation to promote a urine output of more than 0.5 mL/kg/h. They also received a single dose of 2G ceftriaxone intravenously and were investigated by ascertaining a full blood count, urea, and electrolyte levels. The study focused on both emergency and elective abdominal surgeries, excluding obstetric and gynaecological operations.

We excluded patients with organ dysfunction, such as renal impairment, and those who had undergone failed resuscitation for surgery. The study specifically concentrated on cases related to general surgery.

### Data collection

Data was collected by trained research staff and recorded on standardized forms. The following data was collected for each participant: demographic information (age, gender, and location, telephone contact), surgical indication, surgical procedure, preoperative health status, postoperative morbidity and mortality, and length of hospital stay. These data were collected before the surgical procedure and then again at regular intervals for the duration of the hospital stay and after discharge.

### Statistical analysis

The study used STATA/SE14.2 statistical package to analyze data. Descriptive statistics were used to summarize the demographic and clinical characteristics of the participants. Mortality rate and rate of surgical site infections were calculated as the number of deaths or infections divided by the number of participants. The length of hospital stay was calculated as the number of days from the surgical procedure to discharge. Independent samples t-tests were used to compare the mean length of hospital stay between emergency and elective surgery patients. Univariate logistic regression analysis was employed to assess the disparity in the length of hospital stay between elective and emergency patients and to explore the association between age and mortality. Univariate and multivariate logistic regression analyses were used to calculate factors associated with mortality. All statistical tests were performed at a significance level of *p* < 0.05.

### Ethical considerations

The study was approved by the Institutional Review Board of Lacor Hospital.

Informed consent was obtained from each participant before enrollment in the study.

The confidentiality of participant information was maintained throughout the study.

## Results

In this cohort study, a total of 128 patients presented with abdominal signs that required an abdominal operation and the baseline clinical characteristics are shown in Table 1. However, four patients presented with irreversible shock and additional abdominal symptoms, which made it impossible for them to undergo surgery. These four patients were excluded from the study. The remaining 124 patients who underwent surgery were included in the study, and none of them were lost to follow-up. The majority of the patients were male, accounting for 81/124 (65.9%) of the total number of enrolled patients. The average age of the patients was 35.07 ± 17.14 standard deviation (SD) years. The most prevalent symptom reported among the patients was abdominal pain, which was observed in 111 (89.6%) of the patients. A significant number of the patients had undergone surgery before being referred to our facility from other health centres within and outside of Gulu District. Out of the total of 124 patients were admitted to Lacor Hospital, out of which six [6] had undergone surgery at a different medical facility. At Lacor Hospital, four [4] patients required exploratory abdominal surgery with resection and anastomosis for small bowel perforation. Additionally, one patient underwent an omentopexy procedure, and another patient underwent a trauma abdominal surgery for splenic injury/hemoperitoneum. The majority of the patients, 107/124 (86.3%), did not have any underlying comorbidities. However, among those who did have comorbidities, the most common were human immunodeficiency virus (HIV) in 9 (7.3%), hypertension in 4 (3.2%), hepatitis-B in 2 (1.6%), and asthma in 1(0.8%) of the patients. Overall, the most frequent indication for surgery was nontraumatic gastroduodenal perforation in 15 (12.1%) of the patients, followed by splenic trauma in 14 (11.3%). The mean postoperative time to complication was 5.78 (± 3.48 SD) days.

**Table 1:** Baseline clinical characteristics of the participants

Variables	*n* (%)
Gender	
Male Female	82 (65.9) 42 (34.1)
Presence of underlying medical diseases	
Yes No	17 (13.7) 107(86.3)
Shock at presentation	
Yes No	6 (4.8) 118 (95.2)
Renal function test	
Normal Abnormal	119 (92.7) 9 (7.3)
Presence of pneumoperitoneum	
Present Absent	17 (13.7) 107 (86.3)
Anaemia	
Present Absent	7(5.6) 117 (94.4)
Continued post-operative adrenaline infusion	
Yes No	25 (20.2) 99 (79.9)
Category of operation	
Emergency Elective	72 (58.1) 52 (41.9)
Trauma history	
Yes No	21 (19.8) 85 (80.2)
Assisted ventilation	
Yes No	5 (4.0) 119 (96.0)
Presence of surgical site infection	
Yes No	9 (7.3) 115 (92.7)
American society of anesthesiologist classification	
I II III IV	20 (17.1) 41 (35.0) 48 (41.0) 8 (6.8)

**Fig. 1:**
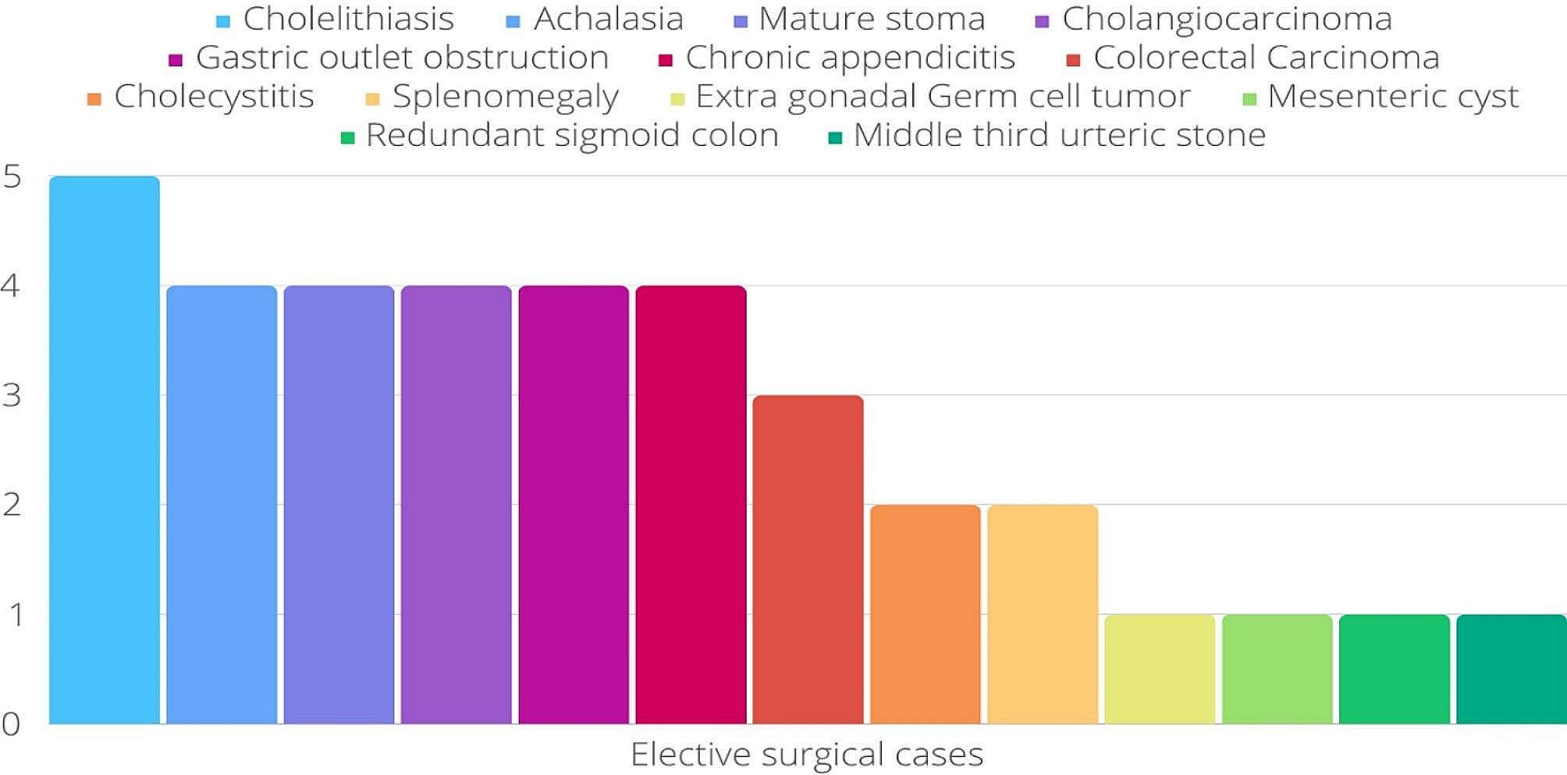
Bar chart showing the different indications elective abdominal surgery

A range of elective surgeries were conducted, with cholelithiasis as the most prevalent indication, accounting for 4.0% of all cases. Other frequent indications included achalasia (3.2%), mature stoma (3.2%), cholangiocarcinoma (3.2%), chronic appendicitis (3.2%), gastric outlet obstruction (3.2%), colorectal carcinoma (2.4%), cholecystitis (1.6%), splenomegaly (1.6%), and various other indications, each contributing 0.8% to all cases. These are visually represented in a Bar chart (Fig. 1).

**Fig. 2:**
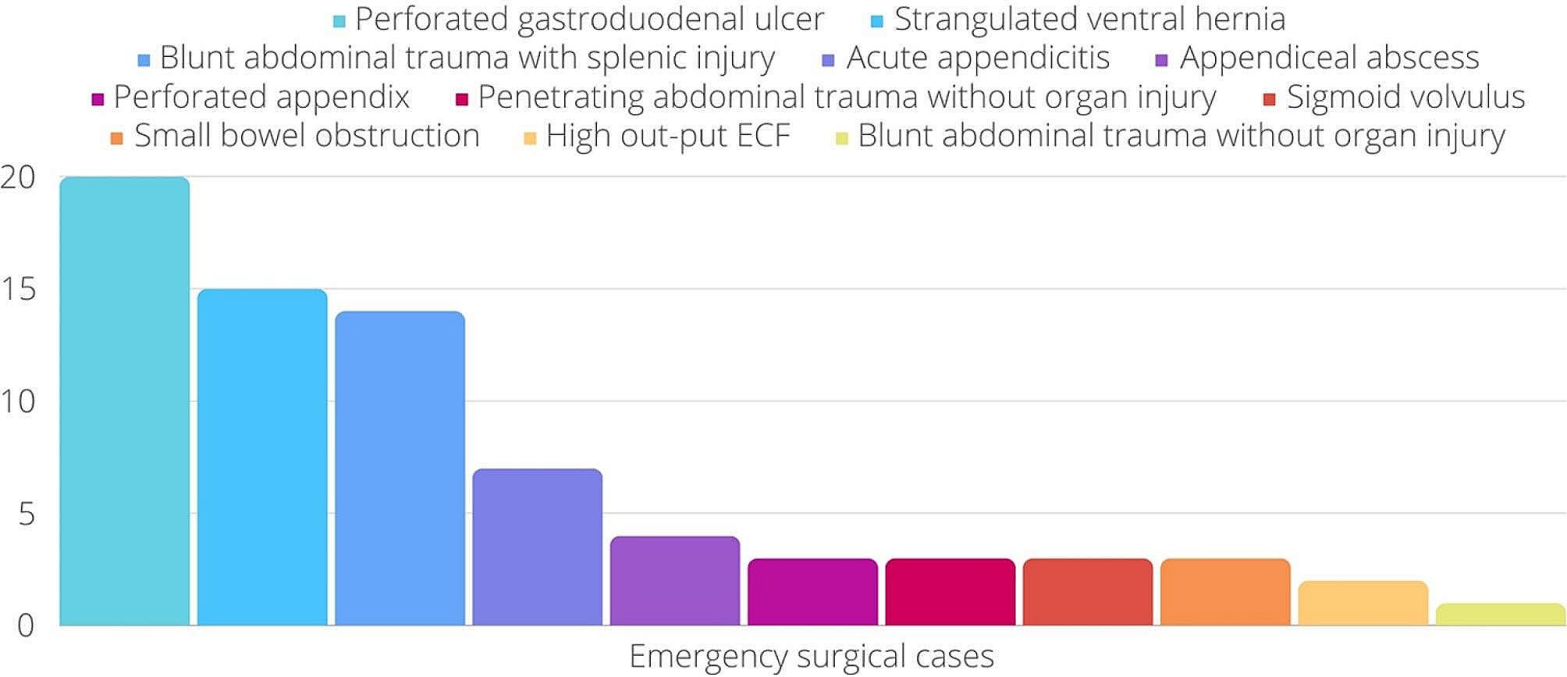
The different indications for emergency abdominal surgery

Emergency surgeries were predominantly driven by perforated gastroduodenal ulcers, constituting 16.1% of cases, followed by strangulated ventral hernia (12.1%) and blunt abdominal trauma with splenic injury (11.3%). Other notable indications included acute appendicitis (9.3%), appendicular abscess (5.3%), and perforated appendix (5.3%). Various conditions such as penetrating abdominal injury, sigmoid volvulus, enterocutaneous fistula, small bowel obstruction, and blunt abdominal injury without organ injury each accounted for 1–3 patients. These key features are visually represented in a bar chart (Fig. 2).

**Table 2:** Short term morbidity following abdominal surgery

Complications	Frequency (*n*)	(%)
Surgical site infection	10	(45.5)
Severe sepsis	4	(18.2)
Septic shock	3	(13.6)
High out-put enterocutaneous fistula/Anastomotic leak	2	(9.1)
Intra-abdominal abscess	1	(4.6)
Burst abdomen	1	(4.6)
Surgical site bleeding	1	(4.6)

After surgery, 17.7% of patients experienced complications, with surgical site infection being the most common at 45.5% (Table 2). Preoperative treatment with 2gm of cefazolin was given to all patients, regardless of whether it was an emergency operation. There was a significant difference in morbidity rate between emergency and electively operated patients (Fischer’s exact *p* < 0.001).

**Fig. 3:**
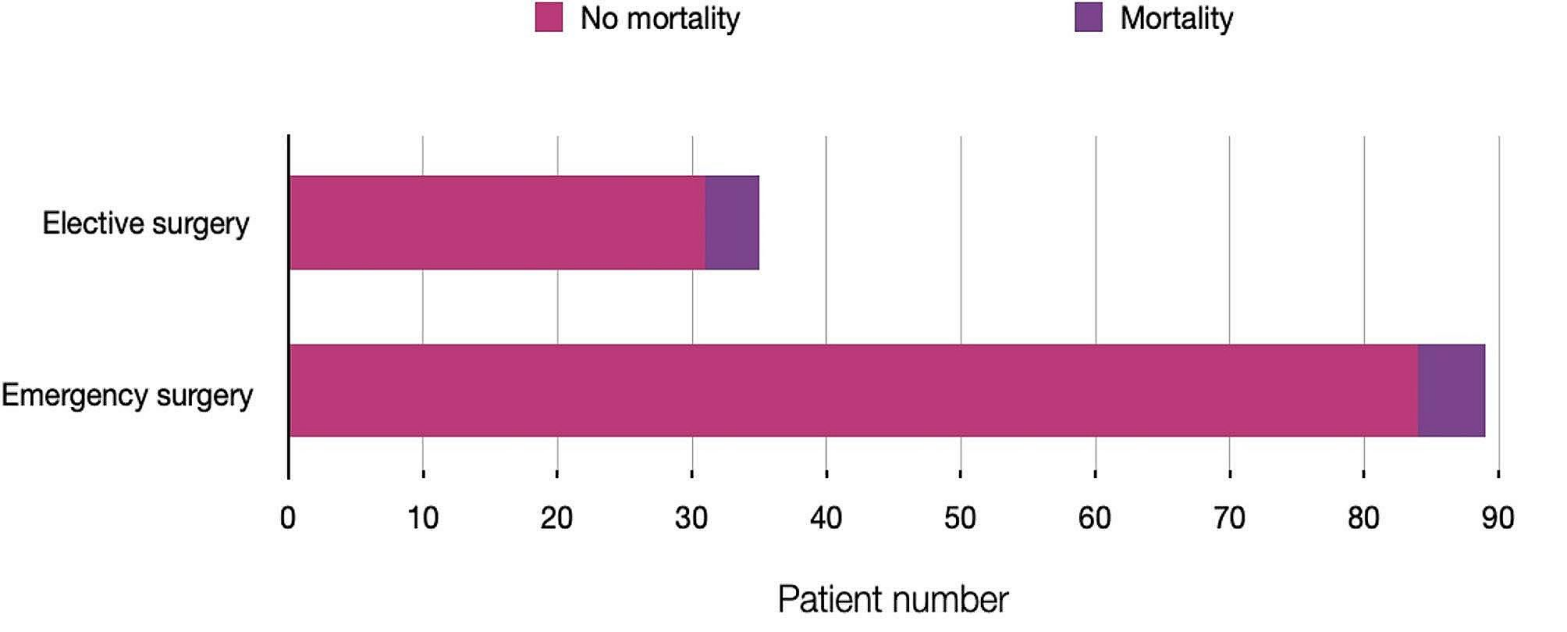
Shows the total number of elective and emergency cases and the mortality

\.

**Fig. 4:**
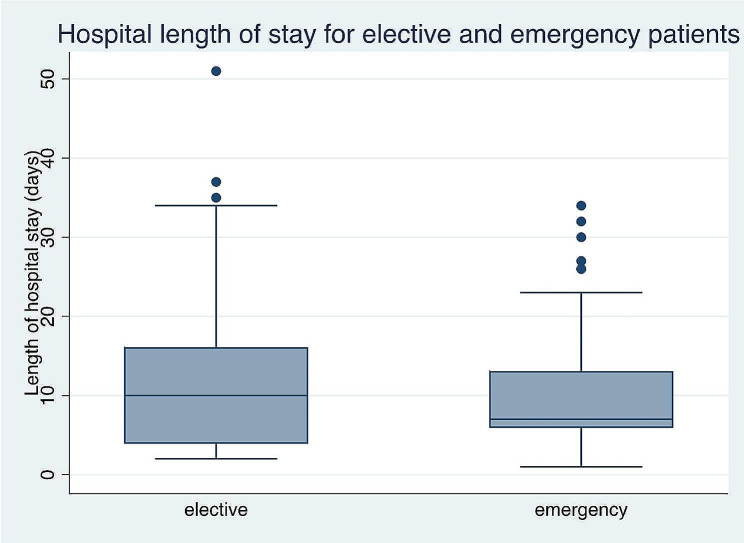
Box-whisker plot showing the length of hospital stay for the elective and emergency operated patients

Of the patients, 35 (28.2%) underwent elective surgery and 89 (71.8%) underwent emergency surgery (Fig. 3). The overall 30-day postoperative mortality rate was 9 (7.3%), with 4 (11.4%) patients undergoing elective surgery and 5 (5.6%)undergoing emergent surgery. The difference in the operative mortality rate between the emergency and elective patients Fischer’s exact test was 0.27. The average hospital stay(Fig. 4) was 11.5 (±9.9 SD) days, with elective patients staying for an average of 14.8 (±10.0 SD) days and emergency patients staying for an average of 10.2 (±8.6 SD) days and COR 0.95, 95% CI 0.91 to 0.99; *P* = 0.02 (Fig. 3).

**Table 3:** Univariate Logistic regression analysis showing factors associated with mortality following abdominal surgery in lacor hospital

Variables		OUTCOME		Crude Odds ratio (COR)	*P*-value	95% Confidence interval (ci)
		Mortality n (%)	No-mortality n (%)			
Gender				1.03	0.97	0.24–4.33
	Male	6 (19.4)	75 (60.5)			
	Female	3 (2.4)	39 (31.5)			
Co-morbidity				1.90	0.45	0.36–10.04
	Yes	2 (1.6)	15 (12.1)			
	No	7 (5.6)	100 (80.6)			
Preoperative hypotension				45.20	< 0.001	6.63-308.07
	Yes	4 (3.2)	2 (1.6)			
	No	5 (4.0)	113 (91.1)			
Impaired renal function				17.60	< 0.001	3.58–86.41
	No	5 (4.0)	110 (88.7)			
	Yes	4 (3.2)	5 (4.0)			
Pneumoperitoneum				0.77	0.814	0.09–6.61
	Present	1 (0.8)	16 (12.9)			
	Absent	8 (6.5)	99 (79.8)			
American society of anesthesiologist classification				1.23	0.768	0.31–4.81
	i	1 (0.8)	19 (15.3)			
	ii	3 (2.4)	38 (30.6)			
	iii	4 (3.2)	44 (35.5)			
	iv	1 (0.8)	7 (5.6)			
Surgery category				0.46	0.271	0.12–1.83
	Elective	4 (3.2)	45 (36.3)			
	Emergency	5 (4.0)	69 (55.6)			
Post operative assisted ventilation status				91.20	< 0.001	8.55–972.91
	Yes	4 (3.2)	1 (0.8)			
	No	5 (4.0)	114 (91.9)			
Post-operative adrenaline infusion				5.94	0.013	1.46–24.09
	Yes	1 (0.8)	12 (9.7)			
	No	4 (3.2)	95 (76.6)			

In this study, preoperative hypotension (*P* < 0.001), impaired renal function (*P* < 0.001), postoperative assisted ventilation (*P* < 0.001) and postoperative use of adrenaline (*P* = 0.013) were identified as factors associated with mortality (Table 3). Patient age had an odds ratio (OR) of 1.04 (*p* = 0.067) and a 95% confidence interval (0.998, 1.075).

**Table 4:** Multivariate logistic regression analysis showing factors associated with mortality following abdominal surgery in lacor hospital

Variables	Adjusted Odds Ratio (AOR)	*p*-value	95% Confidence interval (ci)
Preoperative hypotension	15.22	0.07	0.83–278.86
Deranged renal function test	14.71	0.03	1.38–156.53
Post operative assisted ventilation status	100.81	0.002	5.19–1956.92
Post-operative adrenaline infusion	2.81	0.38	0.28–28.32

In multivariate analysis (Table 4), the associated factor of mortality was post-operative assisted ventilation, AOR = 100.81, *p*-value = 0.002, 95% c.i 5.19–1956.92 and deranged renal function test, AOR = 14.71, *p*-value = 0.03, c.i 1.38–156.53. Preoperative hypotension and post-operative adrenaline infusion are confounders.

**Fig. 5:**
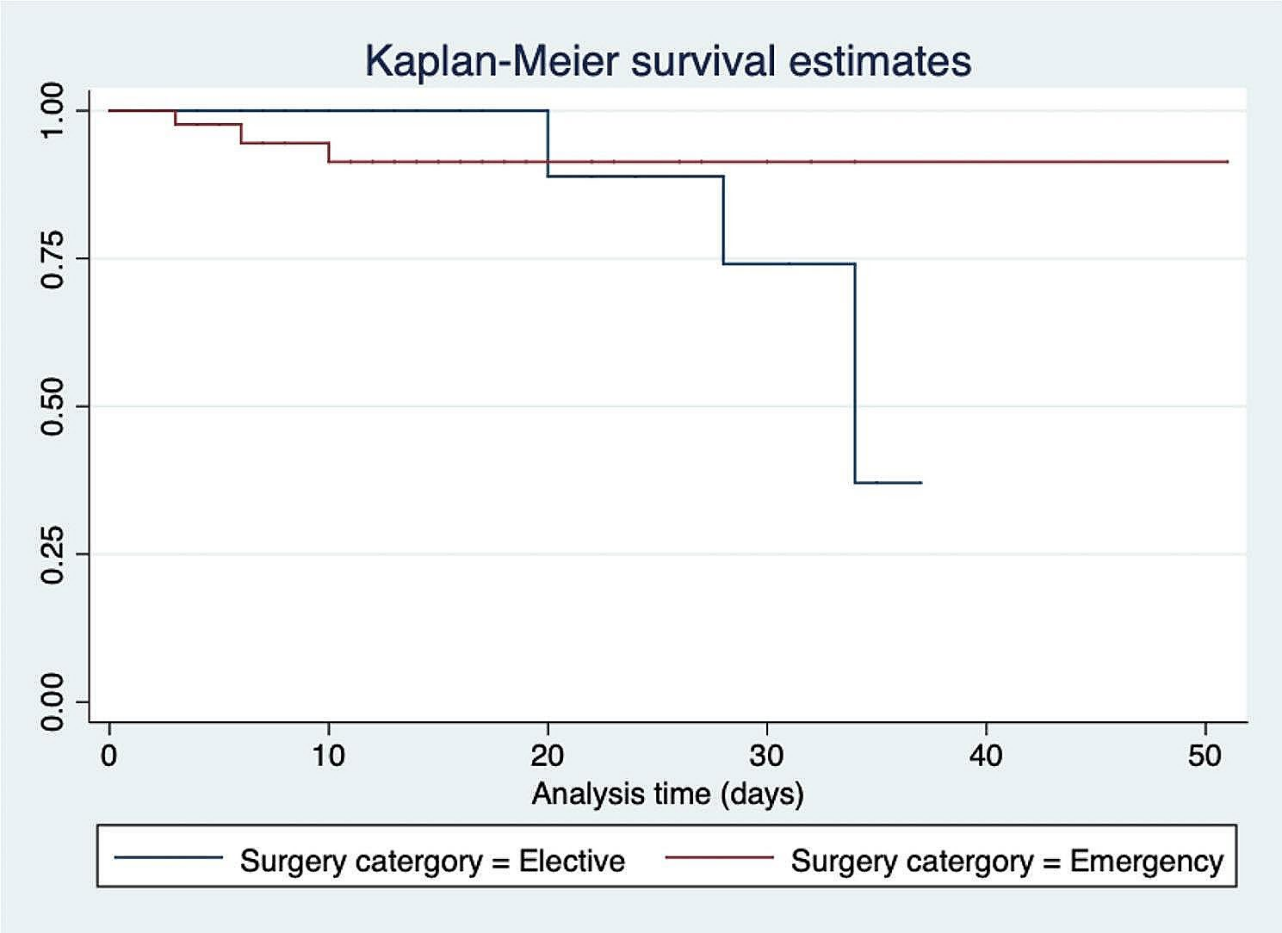
Kaplan-Meier curves showing in-hospital survival following abdominal surgery

The Kaplan-Meier plot (Fig. 5) shows the estimated survival function for each group of patients. The x-axis represents the time from surgery, and the y-axis represents the probability of survival. The graph shows how the probability of survival changes over time for each group. The vertical lines on the graph represent censored observations. The log-rank test (*chi*^*2*^ *= 0.00* and *P-value = 0.99) revealed* no significant difference in survival between patients who underwent elective surgery and those who underwent emergency surgery.

## Discussion

Our study aimed to explore the demographics, indications for surgery, morbidity, and mortality of patients undergoing surgical procedures at Lacor Hospital in Uganda. The study findings indicate that male patients overwhelmingly accounted for the majority of those undergoing general surgical procedures in the given setting. This result is consistent with prior research demonstrating that males are more likely to undergo such procedures [9, 10]. Although sex did not significantly affect the mortality rate after surgery (*P-value* = 0.957), the mean age of patients was lower than that in other studies, possibly due to variations in surgical indications in the study population [5, 21]. Our study still demonstrated that the most frequent indication for surgery is peritonitis caused by gastroduodenal perforation, as previously reported [8–11]. This finding aligns with a prior publication reporting the prevalence of duodenal ulcer (14.8%) and gastritis (12.6%) prevalence in the same area [22]. These consistent findings suggest a potential link between these diagnoses and the observed increase in perforations. However, this differs from other published studies demonstrating that causes of peritonitis other than gastroduodenal perforations are most common indications for abdominal surgery [7, 23, 24].

Compared with elective surgery, emergency surgery is associated with a much longer hospital length of stay and a greater complication rate [8, 25]. The duration of hospitalization for surgical patients can be influenced by multiple factors such as the type of surgery, the overall health status of the patient, and availability of medical resources [18, 19]. In low-income countries such as Uganda, the limited availability of medical resources, such as intensive care units and rehabilitation facilities, can affect the length of stay for both emergency and elective surgery patients. However, this study which was conducted at Lacor Hospital revealed no significant difference in the length of hospital stay between emergency and elective abdominal surgery patients. We believe that the lack of variation in the length of hospital stay can be attributed to the fact that both groups underwent similar incisions on the abdominal wall. Consequently, patients are discharged home only after immediate complications, such as wound infections, have been ruled out and skin sutures removed. Additionally, there is no intermediary nursing home available in the country to transition patients to. Therefore, both groups essentially require a comparable duration of hospitalization. This study also revealed a significant decrease in the rate of surgical site infections (SSIs) compared to a prior study conducted at the same hospital [9] with a rate of 7.3% in the current study. Emergency surgeries, driven by conditions like GI perforations and strangulated hernias, result in higher rates of surgical site infections and sepsis due to the creation of class 3 wounds, which have infection rates of 15-30%. Elective surgeries, with class 1 and class 2 wounds, generally have lower infection rates. The urgency and nature of emergency cases contribute to this disparity in complication rates [26, 27]. Furthermore, the mortality rate was significantly lower than that in a previous study, with rates of 7.5% and 7.0% for emergency and elective surgeries, respectively, though with no statistically significant difference (*P* = 0.891). These results differ significantly from those of previous research, which showed that emergency surgery patients had a greater risk of mortality than did those undergoing elective procedures [8, 9, 28], The presence of an infection prevention committee in the hospital could account for the observed trends. The lack of discernible differences in mortality outcomes within this study may be attributed to advancements in surgical techniques, enhanced infection control measures, and more efficient management of postoperative complications. These improvements over time could have contributed to a reduction in the overall mortality risk for emergency surgery patients, potentially narrowing the gap between their outcomes and those of elective surgery patients. Additionally, the specific patient population with a lower mean age, and a fairly well-equipped hospital facility with clear working systems where there is no delay in attending to patients could have influenced these findings.

## Conclusion

Cholelithiasis was the most common reason for elective surgeries, while gastroduodenal perforation was more common in emergency cases. The length of hospital stay and mortality rates were not significantly different between emergency and elective surgeries. However, emergency surgeries had higher complication rates, with surgical site infection being the most common morbidity. Factors such as deranged renal function and postoperative assisted ventilation status were associated with adverse outcomes.

## Data Availability

The datasets used and/or analyzed during the current study are available from the corresponding author upon reasonable request.
